# Low Magnesium Concentration Enforces Bone Calcium Deposition Irrespective of 1,25-Dihydroxyvitamin D_3_ Concentration

**DOI:** 10.3390/ijms24108679

**Published:** 2023-05-12

**Authors:** Usman Rashid, Sandra K. Becker, Gerhard Sponder, Susanne Trappe, Mansur A. Sandhu, Jörg R. Aschenbach

**Affiliations:** 1 Department of Clinical Studies, Faculty of Veterinary and Animal Sciences, PMAS-Arid Agriculture University, Rawalpindi 46300, Pakistan; usmanrashid@uaar.edu.pk; 2 Institute of Veterinary Physiology, Department of Veterinary Medicine, Freie Universität Berlin, 14163 Berlin, Germany; sandra.becker@health-and-medical-university.de (S.K.B.); gerhard.sponder@genequine.com (G.S.); susanne.trappe@fu-berlin.de (S.T.); 3 Department of Veterinary Biomedical Sciences, Faculty of Veterinary and Animal Sciences, PMAS-Arid Agriculture University, Rawalpindi 46300, Pakistan

**Keywords:** magnesium, vitamin D_3_, cattle, magnesium-responsive genes, vitamin D-responsive genes, osteoblast, osteogenesis, mesenchymal stem cells

## Abstract

Efficient coordination between Mg^2+^ and vitamin D maintains adequate Ca^2+^ levels during lactation. This study explored the possible interaction between Mg^2+^ (0.3, 0.8, and 3 mM) and 1,25-dihydroxyvitamin D_3_ (1,25D; 0.05 and 5 nM) during osteogenesis using bovine mesenchymal stem cells. After 21 days, differentiated osteocytes were subjected to OsteoImage analysis, alkaline phosphatase (ALP) activity measurements, and immunocytochemistry of NT5E, ENG (endoglin), SP7 (osterix), SPP1 (osteopontin), and the *BGLAP* gene product osteocalcin. The mRNA expression of *NT5E*, *THY1*, *ENG*, *SP7*, *BGLAP*, *CYP24A1*, *VDR*, *SLC41A1*, *SLC41A2*, *SLC41A3*, *TRPM6*, *TRPM7*, and *NIPA1* was also assessed. Reducing the Mg^2+^ concentration in the medium increased the accumulation of mineral hydroxyapatite and ALP activity. There was no change in the immunocytochemical localization of stem cell markers. Expression of *CYP24A1* was higher in all groups receiving 5 nM 1,25D. There were tendencies for higher mRNA abundance of *THY1*, *BGLAP*, and *NIPA1* in cells receiving 0.3 mM Mg^2+^ and 5 nM 1,25D. In conclusion, low levels of Mg^2+^ greatly enhanced the deposition of bone hydroxyapatite matrix. The effect of Mg^2+^ was not modulated by 1,25D, although the expression of certain genes (including *BGLAP*) tended to be increased by the combination of low Mg^2+^ and high 1,25D concentrations.

## 1. Introduction

Minerals are vital components of animals’ health and contribute to various physiological processes. Imbalanced mineral content in the body can lead to various health problems. In mammals, pregnancy and the postpartum period are associated with important changes. In particular, high-yielding dairy cows have an increased demand for Ca^2+^ to support milk production after calving. Consequently, circulating levels of Ca^2+^ are often reduced and cows suffer from subclinical (<2 mM or 8 mg/dL) or clinical hypocalcemia (mostly <1.5 mM or 6 mg/dL total calcium in blood plasma) [1]. Hypocalcemia in dairy cows has been associated with several other disorders, namely ketosis, displaced abomasum, and reproductive problems [2]. Mg^2+^ deficiency is known to contribute to the development and severity of hypocalcemia, suggesting a link between hypocalcemia and parathyroid hormone (PTH) receptor signaling at low Mg^2+^ concentrations [3]. Therefore, Mg^2+^ is often infused with calcium to treat acute hypocalcemia [4,5].

The release of PTH and synthesis of 1,25-dihydroxyvitamin D_3_ (1,25D) from 25-hydroxyvitamin D_3_ are key endocrine events that counteract hypocalcemia, leading to urinary and intestinal Ca^2+^ (re)absorption and bone Ca^2+^ mobilization [6]. One key function of PTH is to stimulate the osteolytic activity in bone, thereby enhancing the reabsorption of Ca^2+^ by pericytes [7]. The role of 1,25D in bone appears to be more complex and less well understood. Very low concentrations of 1,25D are known to stimulate the formation of larger amounts of unmineralized osteoid, whereas normal levels of 1,25D are required to generate adequately mineralized cortical and trabecular bone. Increased levels of 1,25D, by contrast, may negatively impact bone mineralization by combined stimulation of osteoclast activity and decreased mineralization efficiency of late osteoblasts/osteocytes [8]. Interestingly, Lou et al. [9] could not find any effect of 1,25D on mineralization in a human osteocyte model differentiated from mesenchymal stem cells with increasing concentrations of 1,25D from 0.05 nM to 1 nM or 10 nM. However, the same treatment resulted in a moderate increase in alkaline phosphatase (ALP) activity and a huge increase in the expression of the vitamin D-responsive gene *CYP24A1* [9]. Thus, the question arises whether the effect of 1,25D on bone formation would be different at low Mg^2+^ concentrations, where PTH signaling should be compromised and, as such, favor osteogenic differentiation [10]. This question is highly relevant to postpartum dairy cows that are often hypocalcemic and where adequate Mg^2+^ levels have been shown to be critical for optimal PTH function and Ca^2+^ regulation [11].

To assess the role of Mg^2+^ in bone calcification and to evaluate the in vitro dose-dependent interaction of 1,25D and Mg^2+^ during osteogenesis, we used a cell culture model of osteogenic differentiation with bovine mesenchymal stem cells (MSCs). The precise source was adipose-derived stem cells (AD-MSC), which have previously been shown to exhibit multilineage (i.e., adipogenic, osteogenic, and chondrogenic) differentiation capacity during in vitro culture [12,13]. This chosen experimental model mimics the in vivo situation where proliferation and differentiation of MSCs into the osteogenic lineage is critical for maintaining bone health [14].

Similar to previous studies in human stem cells, we used 0.05 nM 1,25D to represent a dose at the lower limit of plasma 1,25D concentrations and compared this with a dose that should elicit maximal 1,25D effects, specifically 5 nM [9]. For comparison, plasma concentrations of 1,25D are expected to range between ~0.04 and 1 nM around calving, with the highest values for paretic cows suffering from severe hypocalcemia [15,16]. The concentrations of Mg^2+^ were chosen based on a similar rationale. A concentration of 0.8 mM represents the lowest physiological plasma concentration of Mg^2+^ [17], whereas 3 mM is a very high concentration that should ensure maximum availability of Mg^2+^ for cellular functions. Furthermore, since the effect of decreased Mg^2+^ availability was the main concern, a concentration of 0.3 mM Mg^2+^ was additionally chosen to represent an extreme but realistic Mg^2+^ plasma concentration in hypomagnesemic dairy cows [18]. Our hypothesis was that low Mg^2+^ levels might facilitate the extraction of Ca^2+^ from blood plasma for deposition in bone, and that this process might be modulated by high 1,25D. To test this hypothesis, the cited concentrations of Mg^2+^ and 1,25D were tested for their efficacy in promoting hydroxyapatite matrix deposition during in vitro osteogenic differentiation of bovine MSCs. In parallel, we assessed the activity of alkaline phosphatase, a key enzyme responsible for calcium trapping in bone, the expression of stem cell and osteogenic markers, and the expression of known magnesium- and vitamin D-responsive genes.

## 2. Results

### 2.1. Role of 1,25D and Magnesium in Hydroxyapatite Deposition

**Figure 1:**
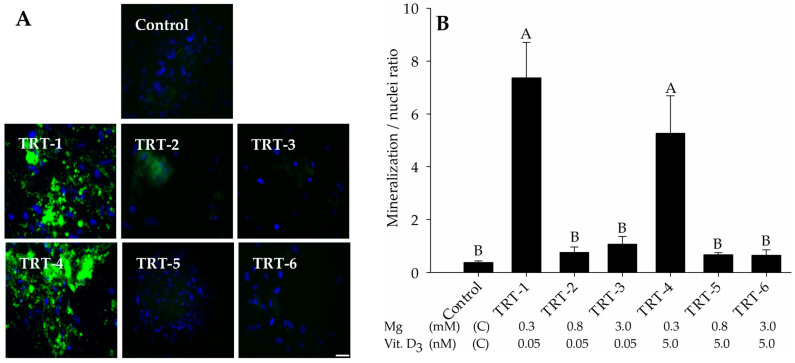
After 21 days, the AD-MSCs were subjected to qualitative and quantitative assessment of osteogenesis. In vitro, osteogenic differentiation was determined by the OsteoImage Mineralization Assay. (**A**) Fluorescence microscopy shows the extracellular hydroxyapatite mineral content imaged at 475/570 nm (excitation/emission; green), whereas the nuclei were stained blue with DAPI, imaged at 358/461 nm. For treatment code, see (**B**). Scale bar 25 µm; 20× objective. (**B**) Quantitative mineralization assessment of developed osteocytes in the different treatment groups. The fluorescence intensity of extracellular hydroxyapatite deposition was divided by DAPI fluorescence to correct for cell density. Data are presented as means ± SEM. ^A, B^ Different superscripts indicate significant differences (*p* < 0.05). Abbreviation; TRT: Treatment.

After 21 days of osteogenesis, the effects of Mg^2+^ and 1,25D were assessed regarding calcium hydroxyapatite deposition in differentiated cells. Treatments were either 0.05 nM 1,25D with 0.3 mM (TRT-1), 0.8 mM (TRT-2), or 3 mM Mg^2+^ (TRT-3), or 5 nM 1,25D with 0.3 mM (TRT-4), 0.8 mM (TRT-5), or 3 mM Mg^2+^ (TRT-6). The deposition of extracellular hydroxyapatite minerals was visualized by fluorescence microscopy using the OsteoImage Mineralization Assay. Our results showed that when cells were treated with the lowest Mg^2+^ concentration, hydroxyapatite matrix was deposited well, whereas matrix deposition was relatively discrete and less intense in all other treatment groups (Figure 1A). The quantitative analysis using a fluorescence plate reader verified the microscopic findings. The lowest level of Mg^2+^ (0.3 mM) showed significantly higher mineralization than the other treatments, irrespective of 1,25D concentration (*p* < 0.001; Figure 1B).

### 2.2. Role of 1,25D and Magnesium in Alkaline Phosphatase Activity

**Figure 2:**
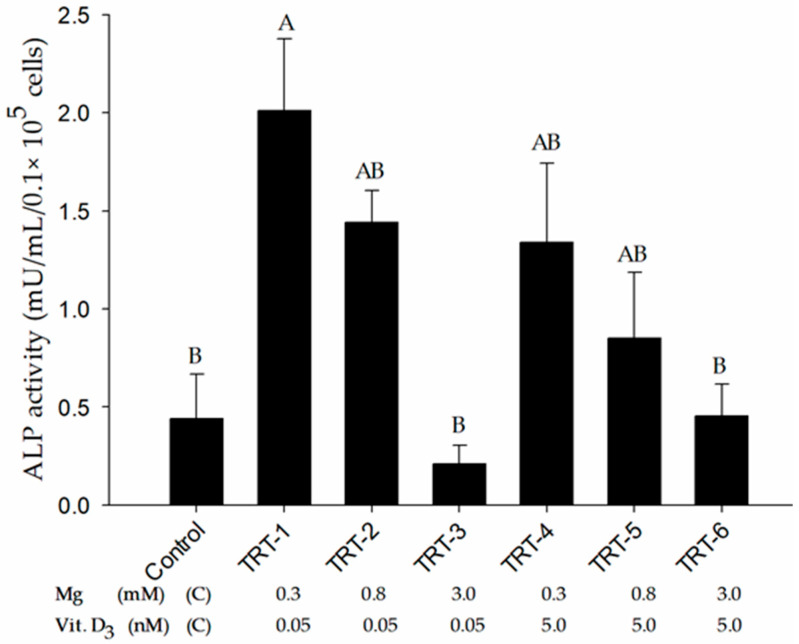
Alkaline phosphatase (ALP) activity of osteogenically differentiated cells was detected with an alkaline phosphatase fluorescence kit. The reading was recorded at 360/440 nm and data are presented as means ± SEM. ^A, B^ Different superscripts indicate significant differences (*p* < 0.05). Abbreviation. TRT: treatment.

The activity of ALP (an early marker of osteogenesis) was assessed after 21 days of incubation in osteogenic medium supplemented with various concentrations of Mg^2+^ and 1,25D. Our results indicated maximal ALP activity in TRT-1 cells with 0.3 mM Mg^2+^ and 0.05 nM 1,25D (Figure 2), which did not differ from that in TRT-2, TRT-4, and TRT-5 cells, but was significantly higher than the control (*p* = 0.011), TRT-3 (*p* < 0.003), and TRT-6 cells (*p* < 0.012). These results supported the assumption that low levels of Mg^2+^ enhance ALP activity in osteogenically differentiated cells independent of 1,25D concentration.

### 2.3. Immunocytochemical Analysis

**Figure 3:**
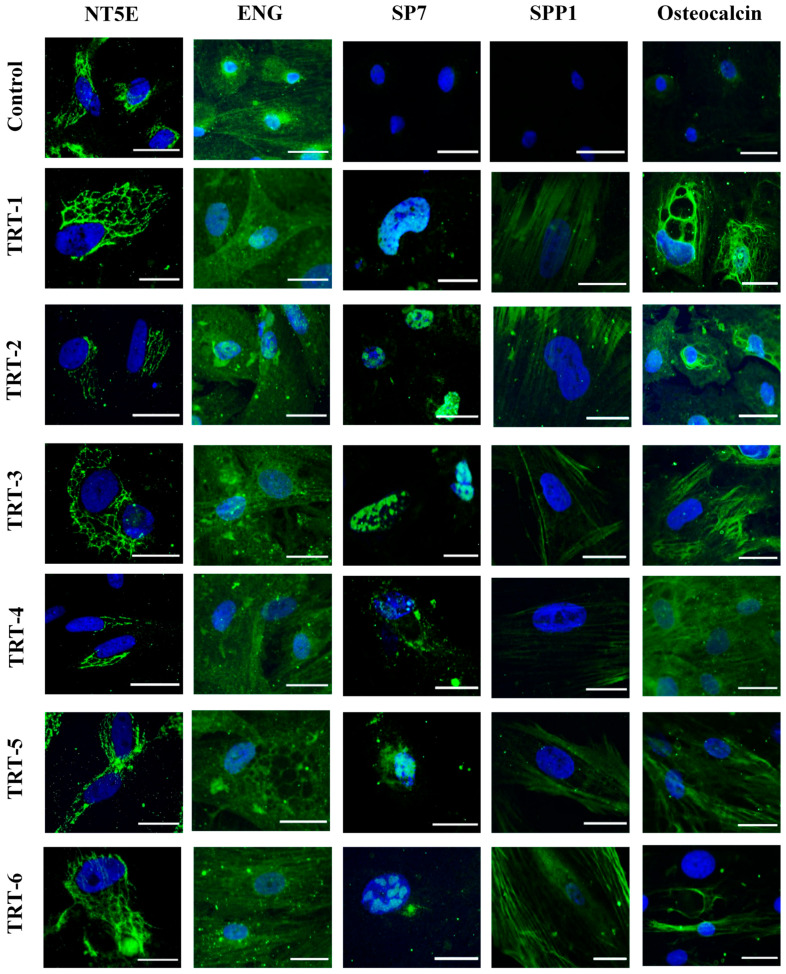
Immunocytochemical analysis of bovine AD-MSCs after 21 days of osteogenesis. Cells were analyzed for the presence of various stem cell and osteogenic markers (green) after culture with various concentrations of Mg^2+^ and 1,25D. Cell nuclei were stained with DAPI (blue). For treatment code, see Figure 1B. Scale bar = 20 µm (63× objective).

To visualize the presence of the stem cell markers NT5E (CD73) and ENG (endoglin, CD105), immunocytochemical analysis was performed on preadipocytes and osteogenically differentiated cells. Immunocytochemistry revealed the presence of NT5E protein in the perinuclear Golgi apparatus in all treatment groups, whereas ENG was widely distributed in the cell membrane regardless of treatment (Figure 3). Expression of the SP7 transcription factor (osterix) was not seen in preadipocytes but was present in all 1,25D-treated cells. The distribution of SP7 expression appeared to be absent in the nuclei of the control group and its intensity varied slightly between treatments. It appeared to be more evenly distributed in the nuclei of the low 1,25D supplementation group, while its presence in the cytoplasm was also evident in the higher 1,25D treatment group. The secreted phosphoprotein 1 (SPP1, osteopontin) and the *BGLAP* gene product osteocalcin are extracellular proteins in bone that did not show any significant expression in preadipocytes in the present study (Figure 3). However, both extracellular proteins formed regular structures in and around cells of all treatment groups.

### 2.4. PCR Analysis

**Figure 4:**
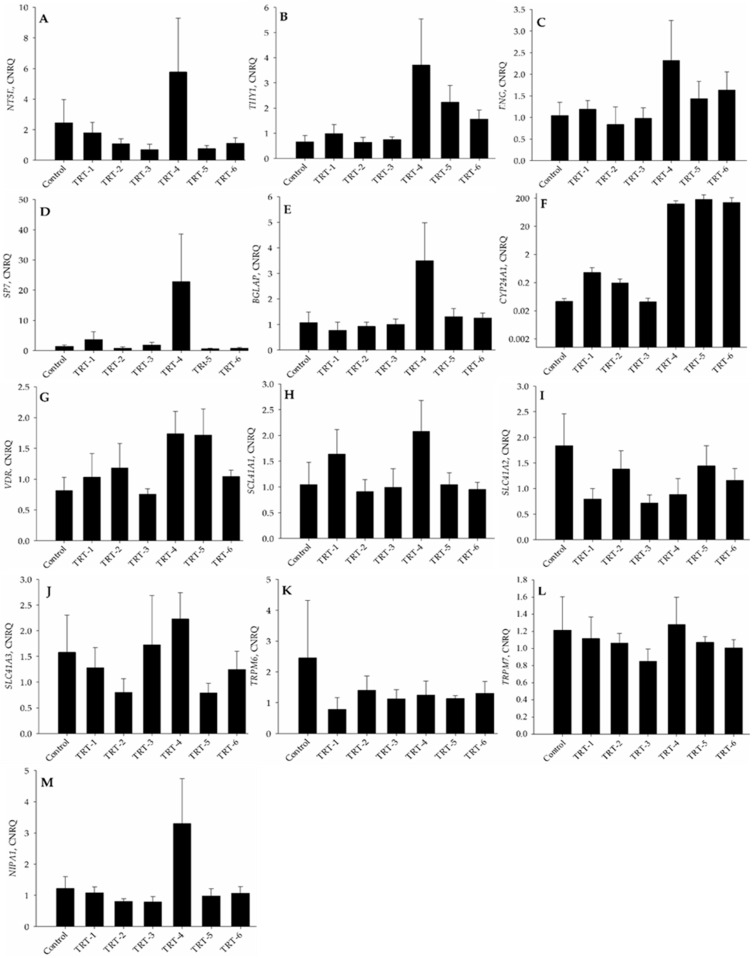
Calibrated normalized relative quantities (CNRQ) of mRNA of the stem cell marker genes (**A**) NT5E (CD73), (**B**) THY1 (CD90), and (**C**) ENG (CD105), the osteogenic genes (**D**) SP7 and (**E**) BGLAP, the vitamin D-related genes (**F**) CYP24A1 and (**G**) VDR, as well as the magnesium-responsive genes (**H**) SLC41A1, (**I**) SLC41A2, (**J**) SLC41A3, (**K**) TRMP6, (**L**) TRMP7, and (**M**) NIPA1 in bovine osteogenically differentiated mesenchymal stem cells. Cells were cultured for 21 days in osteogenic differentiation medium, supplemented with concentrations of Mg^2+^ and 1,25D as indicated in Figure 1B. The Y-axis in panel (**F**) is log-scaled. Results are presented as mean ± SD.

The calibrated normalized relative quantities (CNRQ) of various genes were analyzed after 21 days of osteogenesis. There were no differences in the mRNA expression of the stem cell markers *NT5E* (*p* = 0.241), *THY1* (CD90; *p* = 0.089), and *ENG* (*p* = 0.358), except for a trend towards the highest expression of *THY1* in TRT-4 cells (Figure 4A–C). A similar trend was observed for the osteogenic gene *BGLAP* (*p* = 0.075), but not for *SP7* (*p* = 0.149; Figure 4D,E). The relative expression of the vitamin D-responsive gene *CYP24A1* was higher in TRT-4, TRT-5, and TRT-6 cells than in the cells of other treatment groups (*p* = 0.012; Figure 4F), whereas the mRNA expression of vitamin D receptor (*VDR)* did not differ among groups (*p* = 0.187; Figure 4G). No changes were observed in the expression of most magnesium-responsive genes, i.e., *SLC41A1* (*p* = 0.282; Figure 4H), *SLC41A2* (*p* = 0.298; Figure 4I), *SLC41A3* (*p* = 0.519; Figure 4J), *TRPM6* (*p* = 0.835; Figure 4K), and *TRPM7* (*p* = 0.889; Figure 4L). Only the expression of *NIPA1* tended to be affected by the treatment, with the highest value in TRT-4 cells (*p* = 0.068; Figure 4M).

## 3. Discussion

Magnesium is a mineral of paramount importance for several processes involved in the synthesis and metabolism of vitamin D_3_, including, for example, the enzymatic conversion of 25(OH)D_3_ to 1,25D (active form of vitamin D) [19]. Thus, vitamin D metabolism is part of approximately 300 enzymatic reactions that are dependent on this second most abundant intracellular cation of the body [20]. Adequate levels of Mg^2+^ are required for the activation and normal function of vitamin D. A cohort study in the general human population observed a strong inverse association between vitamin D_3_ and all-cause mortality in participants with high Mg^2+^ intake [21]. This suggests that Mg^2+^ is a master planner in the regulation of vitamin D homeostasis, Ca^2+^ absorption, and overall health.

Based on these premises, our work specifically focused on the interaction of increasing concentrations of magnesium and 1,25D on the osteogenic properties of bovine mesenchymal stem cells. The obtained results showed that extracellular matrix development was significantly higher for the treatment with low Mg^2+^ concentration after 21 days of osteogenesis than for other treatments. Our results thus indicated that lower levels of Mg^2+^ support efficient extracellular mineral deposition rather independent of 1,25D concentration. Alkaline phosphatase is an early osteogenic marker [22] and a prime factor in enhancing the accumulation of extracellular Ca^2+^ through the formation of matrix vesicles at the onset of calcification. Increased extracellular mineral deposition and ALP production in the low-Mg^2+^ groups were coherent with the fact that high extracellular Mg^2+^ levels blocked the mineral matrix deposition and ALP production in human pre-osteoblasts [23]. The reason behind these observations is that excess Mg^2+^ levels alter the concentration of intracellular cations (mainly Ca^2+^) by competing with their transporters. Matrix vesicles are extracellular, membrane-bound, 100 nm in diameter particles located primarily at sites of initial calcification in bone, cartilage, and predentine [24]. These exfoliated matrix vesicles from osteoblasts possess a variety of inorganic phosphates and are strong attractants of Ca^2+^ [25]. Annexin is a Ca^2+^-binding protein involved in the mineralization process whereas ALP functions as an ectoenzyme for the hydrolysis/transphosphorylation of pyrophosphates and ATP for the production of orthophosphates. Upon ALP activation, hydroxyapatite crystals expand in the extracellular environment, leading to Ca^2+^ and PO_4_^3-^ attraction in the extracellular environment and deposition of hydroxyapatite minerals [26,27].

In logical extension of our finding of increased extracellular mineral deposition at low Mg^2+^ concentrations, previous literature had identified decreased hydroxyapatite calcium mineral deposition at very high levels of extracellular Mg^2+^ (>3 mM) in human bone marrow-derived MSCs that were differentiated to osteoblasts. This was paralleled by a decreased frequency and amplitude of calcium oscillations via suppression of spontaneous ATP release and inactivation of purinergic receptors [28,29]. The inhibition of ossification by high concentrations of Mg^2+^ is clinically relevant for surgical osteosynthesis applications. The advantage of Mg^2+^ alloy implants is that they degrade naturally over time and, therefore, do not need to be removed. As a negative side effect, however, bone mineralization is impaired in the surroundings of the degrading Mg^2+^ alloy implant due to locally high Mg^2+^ concentrations [28,29]. 

Previous studies had shown that the addition of 1,25D to the culture medium enhanced the osteogenic differentiation of MSCs because 1,25D plays an important role in calcium–phosphorus homeostasis. Furthermore, 1,25D enhanced the expression of the transcription factors *Runx2* and *Bglap*, together with *Col1A1* (collagen type-1), which are critical for osteogenic differentiation [30]. During in vitro culture, classical MSCs adhere to the surface of plastic culture flasks, express surface markers (NT5E, THY1, and ENG), and differentiate into adipogenic, osteogenic, or chondrogenic lineages [13]. The cells used in the present study met the criteria of MSCs; NT5E was expressed in the Golgi apparatus and there was no change in expression in each treatment group. Reverse transcription quantitative PCR (RT-qPCR) of *NT5E* also showed similar results, where its expression was not significantly different across all treatment groups. Likewise, the qualitative expression of *ENG* remained constant in all treatment groups. In previous studies, lower MSC expression of *ENG* promoted adipogenic and osteogenic differentiation, whereas a high expression of *ENG* was beneficial for chondrogenic differentiation [31,32]. The levels of Mg^2+^ and 1,25D did not induce any significant effect on the expression of these stem cell markers in bovine MSCs. The zinc finger transcription factor SP7 is an important factor in osteogenesis [13] and found primarily in pre-osteoblasts and osteoblasts as an early osteogenic marker [33]. Immunocytochemical analysis revealed that the expression of SP7 accumulated in the nuclei of developing osteoblasts. It is known that ubiquitination of SP7 is critical for osteoblast differentiation and bone mineralization [34]. During in vitro osteogenesis, 1,25D supplementation maximizes the exposure of VDR, which enhances the expression of *Bglap* and bone sialoprotein [35], while Mg^2+^ inversely regulates vascular calcification markers such as *Sp7*, *Spp1*, *Bglap*, and *Runx2* [36]. Our findings suggested that lower levels of Mg^2+^ and a higher level of 1,25D enhance the expression of *SP7*. During the process of osteogenesis, SPP1 is another important protein present in the extracellular matrix. It has a direct role in bone mineralization, wound healing, angiogenesis, cell adhesion, and has a strong affinity for the mineral hydroxyapatite [37]. Osteocalcin is a late differentiation marker that is important for bone mineralization and has a strong affinity for Ca^2+^ and hydroxyapatite substances that promote bone mineralization [38]. The RT-qPCR results of our study showed a trend towards the highest expression of the osteocalcin gene *BGLAP* in the TRT-4 group compared to the rest of the treatment groups. This result was also in line with the physiologic dependence of osteocalcin on vitamin D_3_. In fact, vitamin D_3_ exerts its effects on bone through expression of the osteocalcin gene *BGLAP*. Vitamin D_3_-deficient human patients had lower levels of osteocalcin, suggesting a direct relationship between the two [35,39]. Osteocalcin has been reported to have a strong association with Mg^2+^ supplementation because large amounts of Mg^2+^ inhibit *BGLAP* gene expression [40].

*CYP24A1* and *CYP3A4* hydroxylate 25(OH)D to the inactive form 24,25-dihydroxyvitamin D, which is then converted to water-soluble calcitroic acid for excretion [41]. A strong positive correlation exists between *CYP24A1* and the concentration of 1,25D. The physiologic basis behind these results is to prevent sustained elevation of systemic and cellular levels of 1,25D upon activation of the vitamin D endocrine system [42].

Along with *NIPA1*, the solute carrier family (*SLC41*) genes are an important group of genes that regulate Mg^2+^ transport in and across cells. The NIPA1 protein physically interacts with the type II bone morphogenetic protein (BMP) receptors, inducing its lysosomal degradation and endocytosis, followed by downregulation of BMP signaling [43]. SLC41A1 and SLC41A2 are transmembrane proteins that facilitate cellular Mg^2+^ transport [44], with *SLC41A1* being a Na^+^-dependent Mg^2+^ efflux system at the plasma membrane [45]. In our study, high *SLC41A1* expression was induced by low Mg^2+^ supplementation. SLC41A3 is a mitochondrial protein responsible for the excretion of Mg^2+^ from mitochondria [46]. TRPM6 and TRPM7 are members of the transient receptor potential melastatin family, where TRPM6 is an epithelia-associated protein and TRPM7 is ubiquitous and important for bone health [47]. The current across TRPM7 is stimulated by low intracellular Mg^2+^ levels if they reach 1 mM or less [48]. Expression of *TRMP7* was not significantly altered in the present study although it visually appeared that the expression of *TRPM7* gradually increased with decreasing extracellular Mg^2+^ concentrations irrespective of 1,25D concentration.

In conclusion, the present study shows that low levels of Mg^2+^ promote ALP activity and enhance Ca^2+^ deposition in a bovine bone model of osteogenesis. This supports the view that low levels of Mg^2+^ interfere directly with Ca^2+^ availability in blood plasma and can aggravate hypocalcemia in vivo by enforced Ca^2+^ deposition in bone. This partly opposes the current view in which the aggravating effect of low Mg^2+^ concentration on hypocalcemia is explained solely by the inhibition of Ca^2+^ mobilization due to interference with PTH secretion or signaling to osteoclasts. Interestingly, the effect of low Mg^2+^ concentration on Ca^2+^ deposition appeared to be independent of vitamin D concentration, although vitamin D tended to increase *BGLAP* gene expression at low Mg^2+^ concentrations. Thus, these findings plausibly expand our understanding of the pathophysiology of bovine postparturient hypocalcemia, for which hypomagnesemia has long been known as a predisposing factor. Additionally, these results should also be translatable to humans where a relationship between hypocalcemia and hypomagnesemia is also known but yet explained solely via decreased secretion or action of PTH at low Mg^2+^ concentrations [49,50]. Hypocalcemia in humans is especially prevalent following thyroidectomy. In a large cohort study (126,766 patients), Mg^2+^ disturbances were significantly and independently associated with short- and long-term hypocalcemia after surgery [50]. It would be interesting to test whether this might, in part, be related to increased bone mineral deposition in these patients.

## 4. Materials and Methods

Dulbecco’s phosphate-buffered saline (DPBS), fetal bovine serum (FBS), penicillin–streptomycin, acetic acid, and Dulbecco’s Modified Eagle’s Medium (DMEM) were acquired from Merck Millipore (Darmstadt, Germany). *N*-2-Hydroxyethylpiperazine-*N*′-2-ethane sulfonic acid (HEPES), ascorbic acid, amphotericin B, and trypan blue were purchased from Sigma-Aldrich (Taufkirchen, Germany). 4′,6-Diamidino-2-phenylindole (DAPI) was bought from Roche (Grenzach Wyhlen, Germany). The NucleoSpin^®^ RNA kit was purchased from Macherey-Nagel GmbH & Co. (Düren, Germany). The iScript™ cDNA Synthesis Kit was obtained from Bio-Rad Laboratories GmbH (Munich, Germany). Eurofins Genomics (Ebersberg, Germany) synthesized the primers and probes used in RT-PCR. The cell culture flasks were from Techno Plastic Products (Trasadingen, Switzerland), the 24-well cell culture plates (CytoOne) were from Star lab (Hamburg, Germany), the 96-well plates were sourced from Carl Roth (Karlsruhe, Germany), and the 48-well plates were from Biozym Scientific GmbH (Hessisch Oldendorf, Germany). The Alpha-MEM (Mg^2+^ free) medium was purchased from PAN Biotech (Aidenbach, Germany).

### 4.1. Preadipocytes Isolation and Culture

Bovine preadipocytes isolated from our previous study [51] were cryopreserved at passage (P-2) in DMEM containing 20% FBS and 5% DMSO at −80 °C for the current study. Cells from four calves were thawed and cultured in complete culture medium [DMEM, penicillin/streptomycin (100 U/mL and 100 μg/mL, respectively), amphotericin-B (2.5 μg/mL), 10% FBS] at 37 °C in a humidified environment with 5% CO_2_. After 24 h of cellular attachment, cultures were washed with Dulbecco’s phosphate-buffered saline (without Ca^2+^ and Mg^2+^; DPBS^−/−^) and further cultured in complete culture medium for cell propagation. After reaching 85–90% confluence, cells were trypsinized and the passaging cycle repeated in new T-75 flasks until P-5. At each passage, cells were counted in a Neubauer chamber and cell viability (>95%) was assessed using the trypan blue exclusion assay. In the whole experiment, the culture medium was changed every 48 h if not stated otherwise.

### 4.2. Osteogenic Differentiation

For osteogenesis, a total of 15,000 cells/well were plated in complete medium in 48-well cell culture plates. At 80% confluence, cells were provided with osteogenic medium [α-MEM supplemented with FBS (10%), ascorbate-2-phosphate (50 µM), β-glycerophosphate (10 mM), dexamethasone (100 nM), penicillin–streptomycin (100 U/mL and 100 μg/mL), and amphotericin-B (2.5 μg/mL)], along with three different concentrations of Mg^2+^ (Fluka, Buchs, Switzerland) and two concentrations of 1,25D (Sigma Aldrich, Taufkirchen, Germany), as shown in Table 1. The cells were kept in osteogenic medium for 21 days and the commercially available OsteoImage Mineralization Assay kit (Lonza, Walkersville, MD, USA) was used to determine the development of hydroxyapatite material formation around cells, according to the manufacturer’s instructions. The EnSpire Multimode Plate Reader (PerkinElmer, Waltham, MA, USA) was used to quantify hydroxyapatite formation at excitation/emission wavelengths of 475/530 nm. To obtain the mineralization/nuclei ratio, the fluorescence of DAPI was measured at 358/461 nm and the hydroxyapatite readout was divided by the DAPI nuclear readout (mineralization/nuclei ratio) to correct for varying cell density.

### 4.3. Alkaline Phosphatase Activity

For our experiment, the alkaline phosphatase assay (ALP) was performed using the commercially available fluorescent kit (ab83371; Abcam, Cambridge, UK) according to the manufacturer’s instructions. In biological samples, the detection sensitivity of the kit was ~1 µU. Fluorescence signals were measured using the EnSpire Multimode Plate Reader (excitation/emission = 360/440 nm).

### 4.4. Microscopic Validation of Preadipocyte Differentiation and Hydroxyapatite Formation

Cells were grown on sterilized glass coverslips (6 mm diameter) for immunocytochemistry and processed according to the procedures reported elsewhere [12]. Briefly, the cells were characterized for the presence of surface markers NT5E (CD73, ab137595), ENG (CD105, MA5-11854), SP7 (osterix, ab209484), SPP1 (osteopontin; ab63856), and osteocalcin (ab198228). All primary polyclonal antibodies were raised in rabbits and procured from Abcam (Cambridge, UK), except ENG, which was a mouse monoclonal IgG purchased from Thermo Fisher Scientific (Waltham, MA, USA). For immunocytochemistry, control and osteogenically differentiated MSCs were washed twice with cold DPBS and fixed in 4% Roti-Histofix solution for 30 min after 21 days of culture. The cells were further washed with DPBS for 5 min, treated with 0.3% Triton X-100 for 20 min, and blocked using 5% adult goat serum in PBS for 30 min. Then, cells were incubated with respective primary antibodies in DPBS, i.e., NT5E (diluted 1:100), ENG, SP7, SPP1, and osteocalcin (diluted 1:50), at room temperature for 1 h and further overnight at 4 °C. Subsequently, the coverslips were washed with DPBS and incubated with goat-raised anti-rabbit/mouse secondary antibodies conjugated with Alexa Flour 488 (Thermo Fisher Scientific, Waltham, MA, USA; diluted 1:500) for 45 min at room temperature in the dark. Antibody-conjugated cells were washed thrice with DPBS, incubated with DAPI (0.2 μg/mL) in PBS, and kept in the dark at room temperature for 5 min. The cells were washed thrice with DPBS, mounted on glass slides with Mount Fluor (BioCyc, Potsdam, Germany), and preserved at 4 °C. All negative controls were made by incubating cells with DPBS instead of primary antibody. Stained cells were observed under a Leica DMI 6000B epifluorescence microscope (Leica microsystems, Wetzlar, Germany) at (excitation/emission) 475/570 nm for Alexa Fluor and 358/461 nm for DAPI.

### 4.5. RNA Isolation and RT-qPCR Analysis

After 21 days, cultured AD-MSCs were washed twice with DPBS and trypsinized. Afterwards, cells were centrifuged (300× *g* at 4 °C for 5 min) and stored at −80 °C in RNAlater^®^ (Invitrogen, Carlsbad, CA, USA). Total RNA was extracted using a NucleoSpin^®^ RNA kit (Machery-Nagel GmbH & Co., Düren, Germany) as per the manufacturer’s guidelines. The concentration of isolated RNA was evaluated at 260 nm using a Nano-Photometer (Implen, Munich, Germany), followed by reverse transcription using an iScript cDNA Synthesis Kit (Bio-Rad, Munich, Germany) according to the manufacturer’s instructions. RT-qPCR was carried out in an iCycler (Thermo Scientific, Waltham, MA, USA) using SYBR green master mix (Bio-Rad, Munich, Germany) and gene-specific primer sets for the stem cell markers *NT5E*, *THY1* (*CD90*), and *ENG*, the osteogenic marker genes *BGLAP* and *SP7*, the vitamin D-responsive genes *CYP24A1* and *VDR*, as well as the Mg^2+^-responsive genes *SLC41A1*, *SLC41A2*, *SLC41A3*, *TRPM6*, *TRPM7*, and *NIPA1* (Table 2). All reactions were executed in triplicate, with *GAPDH* as the housekeeping gene. The thermocycler conditions were as follows: initial denaturation at 95 °C for 3 min followed by 40 cycles at 94 °C for 30 s, 58 °C for 1 min, and 72 °C for 30 s. The calibrated normalized relative quantities (CNRQ) of target genes were calculated using the 2^−ΔΔCT^ method. All reactions were run with a negative control without cDNA.

### 4.6. Statistical Analysis

Data were analyzed and graphs were plotted using SigmaPlot software (version 9, Systat Software Inc., San Jose, CA, USA). All data sets presented in this manuscript were obtained from four animals (*n* = 4). For OsteoImage and cellular ALP activity analysis, measurements were pooled arithmetically from three wells of a 48-well plate and two wells of a 24-well cell culture plate, respectively. Each CNRQ value was obtained from triplicate RT-qPCR analysis. Data sets obtained from OsteoImage, ALP activity, and RT-qPCR were analyzed using one-way analysis of variance (ANOVA). If significant, differences between groups were determined by the Holm-Sidak post hoc test. The significance level was set at *p ≤* 0.05. Data are presented as means ± standard errors of the mean.

## Figures and Tables

**Table 1:** Experimental concentrations of magnesium and 1,25-dihydroxyvitamin D_3_ in the osteogenic differentiation media.

Treatments	Mg (mM)	1,25D ^1^ (nM)
Control	0.80	0
TRT-1	0.30	0.05
TRT-2	0.80	0.05
TRT-3	3.00	0.05
TRT-4	0.30	5.00
TRT-5	0.80	5.00
TRT-6	3.00	5.00

^1^ 1,25-dihydroxyvitamin D_3_.

**Table 2:** Primer sequences and amplicon sizes for RT-qPCR assays.

Gene		Sense Primer (5′-3′)	Anti-Sense Primer (3′-5′)	Amplicon Size, bp
Stem cell markers	*NT5E* (*CD73*)	TTTGGAGGCACCTTTGACC	AGAGGCTCATAACTGGGCAC	212
	*THY1* (*CD90*)	CAACTTCACCACCAAGGATG	TCTGGATCAGCAGGCTTATG	140
	*ENG* (*endoglin*, *CD105*)	CCTCAGCGTGAACAAATCC	CGTGAAAGACCAGTTTGGAG	89
Osteogenic genes	*SP7 (osterix)*	TGCTTGAGGAGGAAGCTCAC	TTTGGAGGCCGAAAGGTCAC	160
	*BGLAP* (*osteocalcin)*	GCAAAGGCGCAGCCTTCGTG	AAGCCGATGTGGTCAGCTAG	174
Magnesium-responsive genes	*SLC41A1*	TGGTGTTCCTCTATACCATCAG	TCAAGTACGGGATGGAGAAG	186
	*SLC41A2*	CTGCTTTTAGTGATACCTGGAC	TTCCTTTCCTCCAGAAATGATG	178
	*SLC41A3*	CTTCTGCACTATTTCCAGCAC	TCATCTCCAGGTTGCCCTTC	100
	*TRPM6*	ACAAACCATTCCCTACACTCC	CGTTGTTGTTGTTGTACTTCC	125
	*TRPM7*	ATACAAGAGGGGAGTTACTGG	GGGCCAAAAACCATATCACAG	112
	*NIPA1*	TCCCCGAAATCTGAGAGTGTG	AGAAGATGAGCAGCAGCAGC	115
Vitamin D-responsive genes	*VDR*	TTCGCTCCAACCAGTCCTTC	CTCTTCGTGCAAATTCAGCTTC	166
	*CYP24A1*	CAATTTATCCCGTAATCCCCAC	AGCATATTCCCCCAGAACC	204
Housekeeping gene	*GAPDH*	AAGAAGGTGGTGAAGCAGG	GCATCGAAGGTAGAAGAGTGAG	116

## Data Availability

The datasets analyzed during the current study are available from the corresponding author upon reasonable request.
